# Machine learning in a real-world PFO study: analysis of data from multi-centers in China

**DOI:** 10.1186/s12911-022-02048-5

**Published:** 2022-11-24

**Authors:** Dongling Luo, Ziyang Yang, Gangcheng Zhang, Qunshan Shen, Hongwei Zhang, Junxing Lai, Hui Hu, Jianxin He, Shulin Wu, Caojin Zhang

**Affiliations:** 1 Guangdong Cardiovascular Institute, Guangdong Provincial People’s Hospital, Guangdong Academy of Medical Sciences, 106 Zhongshan 2Nd Road, Guangzhou, 510080 Guangdong China; 2 Zhongnan Hospital of Wuhan University, Wuhan, China; 3 Wuhan Asian Heart Hospital, Wuhan, China; 4 Hubei Huiyi Cardiovascular Center, Enshi, Hubei China; 5 Jiang Men Central Hospital, Jiangmen, Guangdong China; 6 The First People’s Hospital of Foshan, Foshan, Guangdong China; 7 General Hospital of Southern Theatre Command of PLA, Guangzhou, China

**Keywords:** Machine learning, Patent foreman ovale, Device closure, Recurrent stroke, Transient ischemic attack

## Abstract

**Purpose:**

The association of patent foreman ovale (PFO) and cryptogenic stroke has been studied for years. Although device closure overall decreases the risk for recurrent stroke, treatment effects varied across different studies. In this study, we aimed to detect sub-clusters in post-closure PFO patients and identify potential predictors for adverse outcomes.

**Methods:**

We analyzed patients with embolic stroke of undetermined sources and PFO from 7 centers in China. Machine learning and Cox regression analysis were used.

**Results:**

Using unsupervised hierarchical clustering on principal components, two main clusters were identified and a total of 196 patients were included. The average age was 42.7 (12.37) years and 64.80% (127/196) were female. During a median follow-up of 739 days, 12 (6.9%) adverse events happened, including 6 (3.45%) recurrent stroke, 5 (2.87%) transient ischemic attack (TIA) and one death (0.6%). Compared to cluster 1 (*n* = 77, 39.20%), patients in cluster 2 (*n* = 119, 60.71%) were more likely to be male, had higher systolic and diastolic blood pressure, higher body mass index, lower high-density lipoprotein cholesterol and increased proportion of presence of atrial septal aneurysm. Using random forest survival (RFS) analysis, eight top ranking features were selected and used for prediction model construction. As a result, the RFS model outperformed the traditional Cox regression model (C-index: 0.87 vs. 0.54).

**Conclusions:**

There were 2 main clusters in post-closure PFO patients. Traditional cardiovascular profiles remain top ranking predictors for future recurrence of stroke or TIA. However, whether maximizing the management of these factors would provide extra benefits warrants further investigations.

**Supplementary Information:**

The online version contains supplementary material available at 10.1186/s12911-022-02048-5.

## Introduction

Foreman ovale is a bridging structure during embryological development. Normally, this structure closes spontaneously after birth. If it is not the case, a patent channel will be formed and named as patent foreman ovale (PFO), predisposing an increased risk of paradoxical embolisms [1, 2]. The association of PFO and stroke was first proposed in 1988 [3]. Since then, numerous studies, including observational or randomized control trials (RCTs), have shown potential causal effect of PFO on cryptogenic stroke (CS) [4–10]. The prevalence of PFO in general population is around 25%, but reaches up to 40% in CS patients [3].

Although the first three RCTs suggested a neutral effect of PFO closure on stroke prevention [4–6], latest RCTs [7, 9, 10] and updated meta-analyses [11, 12] all supported the benefits of PFO closure. In view of these data, current guidelines have recommended PFO closure in patients with proved PFO-associated stroke (PFO-AS), a new concept created in 2020 [13, 14]. Although device closure on average decreases the risk for recurrent stroke, treatment effects varied substantially across different studies [15].

In recent years, the applications of artificial intelligence or machine learning have shown promising results in health care system. It not only helps the process of data management, but also aids in disease prediction or patient sub-clustering. For example, in a data-driven machine-learning analysis, the authors applied hierarchical k-means clustering algorithm to explore potential sources of embolic stroke [16]. In another study, machine learning was used to automatically discriminate cardioembolic from non-cardioembolic strokes in large datasets [17].

Understanding different causes and classifying strokes based on etiologic subtypes are prerequisites for effective treatments. With the state-of-the-art machine learning, we could easily identify subsets of patients that would benefit most from PFO closure or sustain elevated risk for recurrent stroke. Thus, in this study, we applied unsupervised machine learning to detect sub-clusters in post-closure PFO patients and assess their associated risk with adverse outcomes. As the second aim, we used supervised machine learning to identify potential predictors for adverse outcomes.

## Methods

### Study design and population

The analyzed population was from 7 centers in China, including Guangdong Cardiovascular Institute, Zhongnan Hospital of Wuhan University, Wuhan Asian Heart Hospital, Hubei Huiyi Cardiovascular Center, Jiang Men Central Hospital, the first people's hospital of Foshan and General Hospital of Southern Theatre Command of PLA. Patients with embolic stroke of undetermined sources (ESUS) and PFO were included during June 1st, 2013 and May 31st, 2020. The diagnosis of ESUS was deliberately and systematically assessed by both a neurologist and a cardiologist after excluding the other common etiologies of stroke. PFO was initially discovered by either transthoracic/transesophageal or right heart contrast echocardiography and finally confirmed during cardiac catheterization. Patients with overt alternative causes of their strokes or not receiving PFO closure were excluded. We collected demographic information, laboratory and echocardiographic data for the included subjects.

### Outcome ascertainment

Patients were followed by regular telephone interviews or outpatient examinations. The main outcome in our study was a composite of recurrent ischemic stroke, transient ischemic attack (TIA) or all-cause death. Major bleeding or new-onset atrial fibrillation (AF) was examined as secondary outcomes. Cardiac rhythm was assessed by cardiac auscultation, which was followed by electrocardiography if abnormal auscultation was found. At each telephone interview or outpatient visit, a standardized and validated questionnaire (Questionnaire for verifying stroke-free status) was used to detect potential stroke or TIA.

### Statistical analysis

Student t-test and chi-square test were used for comparisons of differences between groups. Data were shown as mean (SD), median (interquartile range[IQR]) or number (percentage). Two-sided *P* < 0.05 was considered to be significant.

Data pre-processing was conducted before machine learning. The summary for missing data is shown in Additional file 1: eTable1. As suggested by the missing data pattern (Additional file 1: eFigure1), it was considered as random missing data case with no particular trend among all the variables. Missing values were then computed with multiple imputation using R package of “mice”. Thirty-four variables from demographic, laboratory and echocardiographic data were finally included.

We first used principal component analysis (PCA) to reduce the dimensions with the function of FAMD (Factor Analysis of Mixed Data). Next, we applied cluster analysis on the PCA outputs using the function of HCPC (Hierarchical Clustering on Principal Components) in FactoMineR package. The partitioning of the HCPC is performed by cutting the hierarchical tree (dendrogram). To consolidate the final partitioning solution, we further performed k-means clustering. The binary data was treated as numeric values before clustering [18]. The average silhouette of observations for different values of k (1 to 10) were computed. The location of the maximum is considered as the optimal number of clusters. Cox proportional hazards regression was then applied to calculate the hazard ratio (HR) and 95% confidence interval (95%CI) of adverse events by different clusters. Proportional-hazards assumption was tested and no violation was found. To examine potential bias from the imputed datasets, we performed complete case analysis as one of the sensitivity analyses.

In supervised learning, we first used all available variables to construct the random forest survival (RFS) model and accessed the variable importance (VIMP). We then selected the top ranking features to reconstruct the prediction models and assessed the performance using concordance index (C-index) and Brier score (BS). A higher C-index and lower BS suggest a better prediction performance. In addition, we applied supervised self-organizing maps to help visualize features associated with individual cluster within the studied patients. All statistical analyses were performed using R version 4.1.2 or Stata 15.1 (StataCorp/SE, College Station, TX). Detailed descriptions of R source code were disclosed in the supplement.

## Results

**Fig. 1:**
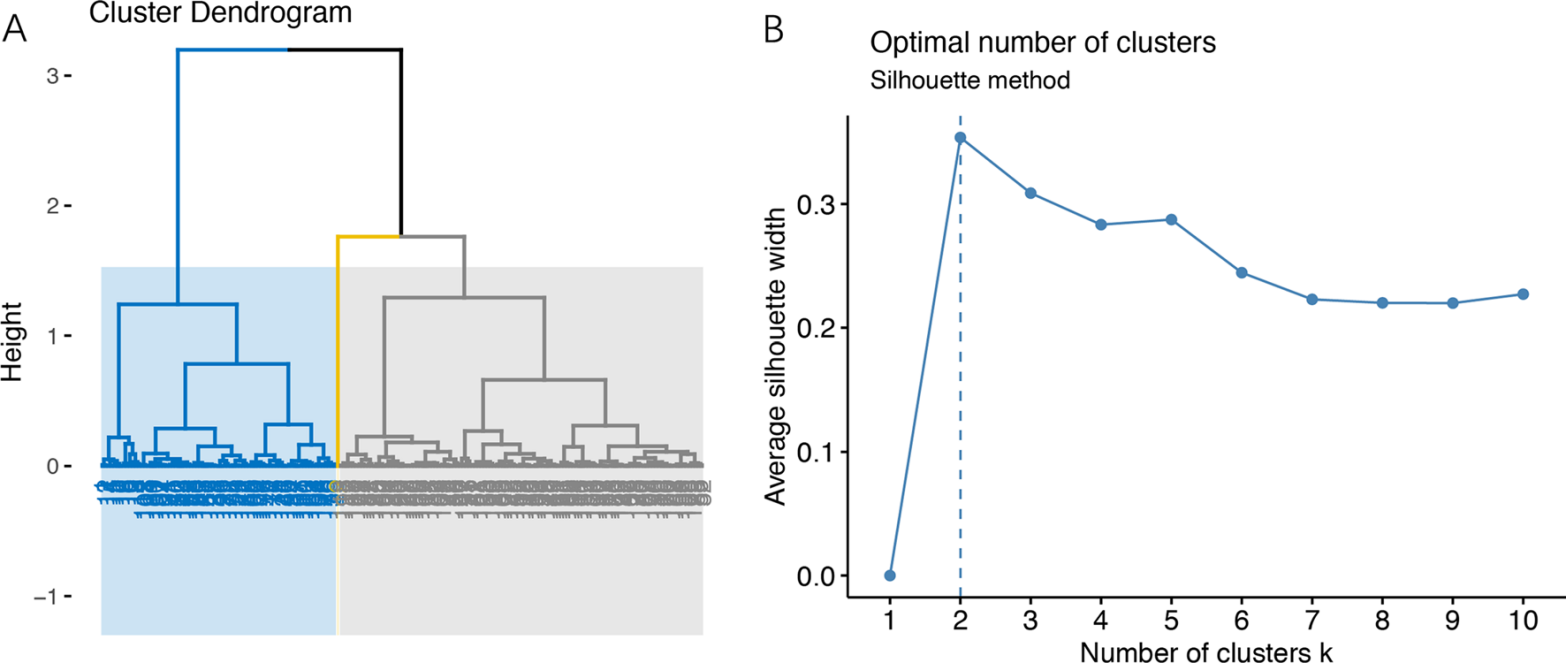
Clusters identified by different methods. **A** Dendrogram from hierarchical clustering on principal components analysis. **B** The average silhouette of observations for different values of k (1 to 10) using k-means clustering analysis. The highest average silhouette was located at k = 2

197 PFO patients receiving percutaneous interventions were initially included in this study. The first 12 principal components with an eigenvalue ≥ 1, which accumulatively counted for 70.62% of the dataset, were used as input for the HCPC method (Additional file 1: eTable 2). Using HCPC, 3 clusters were identified (Fig. 1-A). Since the middle cluster included only 1 patient, we excluded this cluster, leaving a final number of 196 patients for subsequent analysis. Briefly, the average age of the included subjects was 42.7 (12.37) years and 64.80% (127/196) were female. During a median follow-up of 739 (IQR 731–924) days, 22 (11.22%) patients were lost to follow up. A total number of 12 adverse events (12/174, 6.9%) were reported, including 6 recurrent stroke (6/174, 3.45%), 5 TIA (5/174, 2.87%) and one death (1/174, 0.6%). No AF or major bleeding was documented.

**Fig. 2:**
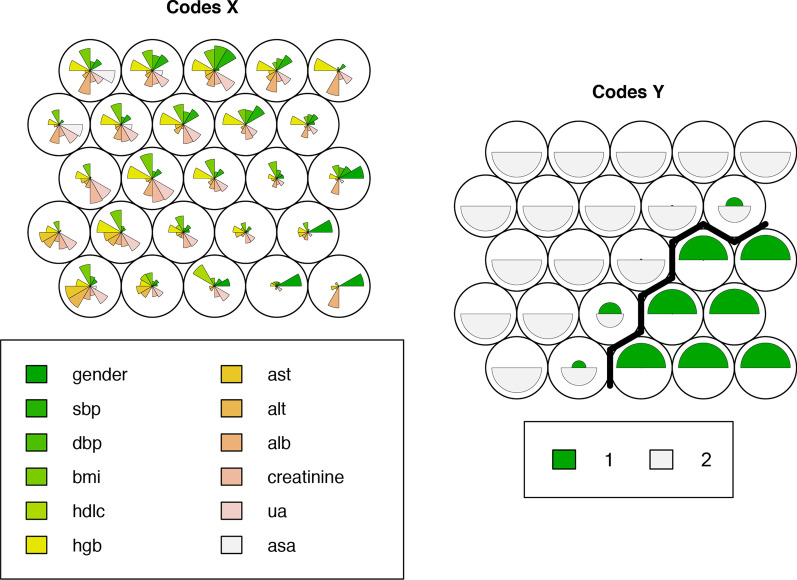
Self-organizing maps supervised by clusters identified by HCPC analysis. HCPC, hierarchical clustering on principal components; sbp, systolic blood pressure; dbp, diastolic blood pressure; bmi, body mass index; hdlc, high-density lipoprotein cholesterol; hgb, hemoglobin; ast, aspartate aminotransferase; alt, alanine aminotransferase; alb, albumin; ua, uric acid, asa, atrial septal aneurysm

**Fig. 3:**
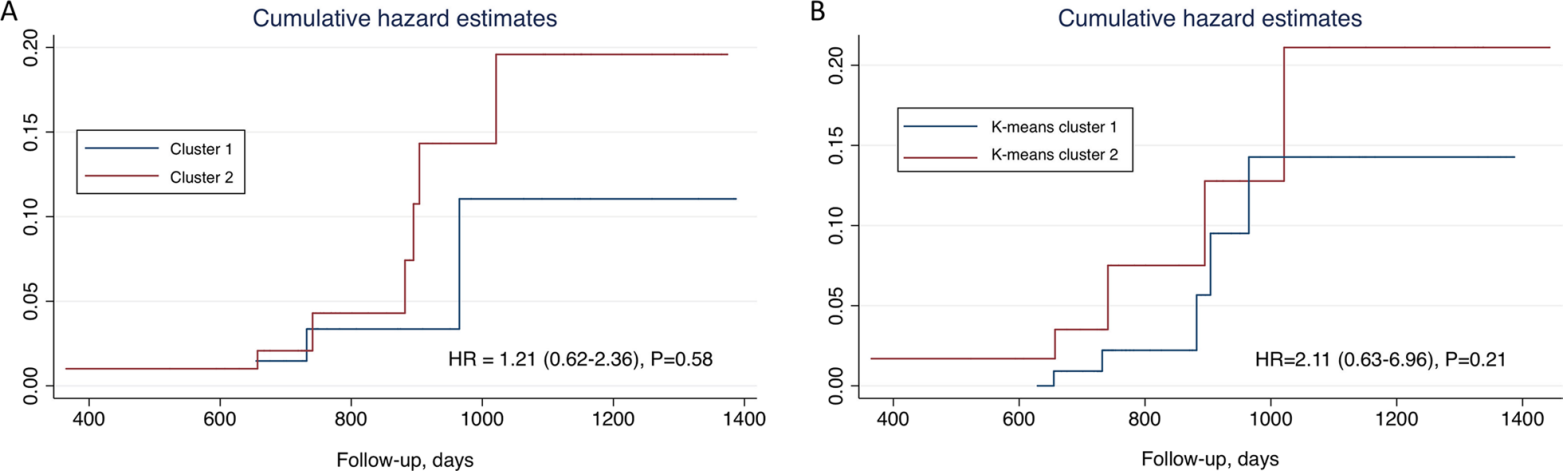
Cumulative hazard estimates of adverse events according to the identified clusters. **A** Clusters from hierarchical clustering on principal components analysis. **B** Clusters from k-means clustering. HR, hazard ratio

Among the analyzed subjects, 77 (39.29%) patients were assigned to cluster 1 and 119 (60.71%) were assigned to cluster 2. Compared to cluster 1, patients in cluster 2 were more likely to be male, had higher systolic and diastolic blood pressure, higher body mass index (BMI), lower high-density lipoprotein cholesterol (HDL-C) and increased proportion of presence of atrial septal aneurysm (ASA). The values of red blood cell, hemoglobin, creatinine, uric acid, left atrium (LA), left ventricular end-diastolic dimension (LVEDD), interventricular septum (IVS) and posterior wall thickness (PW) were also higher in patients of cluster 2. Detailed descriptions of these variables were summarized in Table 1 and vividly visualized in Fig. 2. In Cox regression analysis, patients in cluster 2 tended to have 21% increased hazards for adverse events than those in cluster 1 (HR 1.21, 95%CI 0.62–2.36, *P* = 0.58, Fig. 3-A).

**Table 1:** Baseline characteristics of the study patients according to the clusters (from HCPC)

	Total (*n* = 196)	Cluster 1 (*n* = 77)	Cluster 2 (*n* = 119)	*P* value
Age, years	42.70(12.37)	43.42(11.45)	42.24(12.95)	0.52
Gender, male	**127(64.80%)**	**12(15.58%)**	**115(96.64%)**	** < 0.001**
Han destiny	179(91.79%)	69(90.79%)	110(92.44%)	0.68
Heart rate, bpm	77.30(11.46)	78.60(12.24)	76.45(10.83)	0.20
Systolic BP, mmHg	**121.83(15.64)**	**117.27(16.63)**	**124.78(14.28)**	** < 0.001**
Diastolic BP, mmHg	**76.74(10.78)**	**73.49(9.94)**	**78.85(10.78)**	** < 0.001**
Body mass index, kg/m2	**23.37(3.25)**	**22.25(2.91)**	**24.12(3.26)**	** < 0.001**
History of stroke	120(61.22%)	44(57.14%)	76(63.87%)	0.35
History of TIA	84(42.86%)	36(46.75%)	48(40.34%)	0.38
History of migraine	27(13.78%)	14(18.18%)	13(10.92%)	0.15
*Laboratory data*				
Red blood cell, 10*12/L	**4.58(0.68)**	**4.16(0.52)**	**4.86(0.63)**	** < 0.001**
Hemoglobin, g/dl	**132.92(18.96)**	**116.57(15.09)**	**143.50(12.66)**	** < 0.001**
HCT, L/L	**40.16(5.09)**	**35.96(4.21)**	**42.88(3.52)**	** < 0.001**
APTT, s	35.02(5.70)	34.73(6.24)	35.22(5.32)	0.56
PT, s	13.21(2.82)	13.18(2.97)	13.22(2.73)	0.91
PTA%, %	71.80(42.27)	65.47(43.75)	76.21(40.85)	0.11
Fibrin, mg/dl	3.11(2.06)	3.25(2.36)	3.02(1.82)	0.44
AST, U/L	**23.12(10.33)**	**20.58(9.58)**	**24.75(10.52)**	**0.006**
ALT, U/L	**27.90(22.14)**	**19.06(13.06)**	**33.67(24.84)**	** < 0.001**
Albumin, g/dl	**40.77(4.70)**	**39.91(5.90)**	**41.31(3.69)**	**0.04**
Creatinine, umol/L	**69.62(15.39)**	**58.81(9.91)**	**76.58(14.24)**	** < 0.001**
BUN, mmol/L	4.95(2.73)	5.16(4.10)	4.81(1.20)	0.37
Uric acid, umol/L	**363.32(96.93)**	**289.75(64.70)**	**408.80(84.97)**	** < 0.001**
Fasting blood glucose, mg/dL	5.04(1.25)	5.03(1.18)	5.05(1.30)	0.94
Triglycerides, mg/dL	1.39(0.88)	1.29(1.02)	1.45(0.77)	0.26
Total cholesterol, mg/dL	4.09(1.13)	4.27(1.20)	3.98(1.08)	0.11
LDL-C, mg/dL	2.38(0.84)	2.39(0.83)	2.38(0.85)	> 0.99
HDL-C, mg/dL	**1.21(0.56)**	**1.46(0.77)**	**1.05(0.26)**	** < 0.001**
*Echocardiographic data*				
Presence of atrial septal aneurysm	**17(9.66%)**	**2(2.99%)**	**15(13.76%)**	**0.02**
LA, mm	**32.15(4.08)**	**30.77(3.73)**	**33.03(4.06)**	** < 0.001**
LVEDD, mm	**45.52(3.65)**	**43.79(3.40)**	**46.62(3.38)**	** < 0.001**
LVESD, mm	29.47(4.08)	28.97(4.22)	29.81(3.97)	0.18
IVS, mm	**9.44(1.51)**	**8.67(1.21)**	**9.94(1.48)**	** < 0.001**
PW, mm	**9.23(1.25)**	**8.60(1.13)**	**9.63(1.16)**	** < 0.001**
LVEF, %	65.87(5.49)	65.90(5.31)	65.87(5.49)	0.97
MVE/MVA	1.12(0.34)	1.13(0.35)	1.12(0.34)	0.83
RVEDD, mm	29.28(10.53)	29.36(10.23)	29.22(10.77)	0.94

*p* value < 0.05 are shown in bold

*HCPC* Hierarchical Clustering on Principal Components; *TIA* transient ischemic attack; *AST* Aspartate aminotransferase; *ALT* Alanine aminotransferase; *LDL-C* low-density lipoprotein cholesterol; *HDL-C* high-density lipoprotein cholesterol; *LA* left atrium; *LVEDD* left ventricular end-diastolic dimension; *LVESD* left ventricular end-systolic dimension; *IVS* interventricular septum; *PW* posterior wall; *LVEF* left ventricular ejection fraction; *RVEDD* right ventricular end-diastolic dimension

In k-means clustering analysis, the highest average silhouette was located at k = 2, suggesting 2 as the optimal number of clusters (Fig. 1-B). Detailed descriptions of baseline characteristics across the 2 clusters were summarized in Additional file 1: eTable 3. Generally, the results were similar to what we found from HCPC. Cox regression analysis also suggested that patients in high risk cluster tended to have increased hazards for adverse events (HR 2.11, 95% CI 0.63–6.96, *P* = 0.21, Fig. 3-B). And the high risk cluster was characterized by higher proportion of male gender, higher blood pressure, higher BMI and lower HDL-C. Likewise, the finding from complete case analysis was largely identical to that of the primary analysis, except that the analyzed sample was significantly reduced (Additional file 1: eFigure2 and eTable4).

**Fig. 4:**
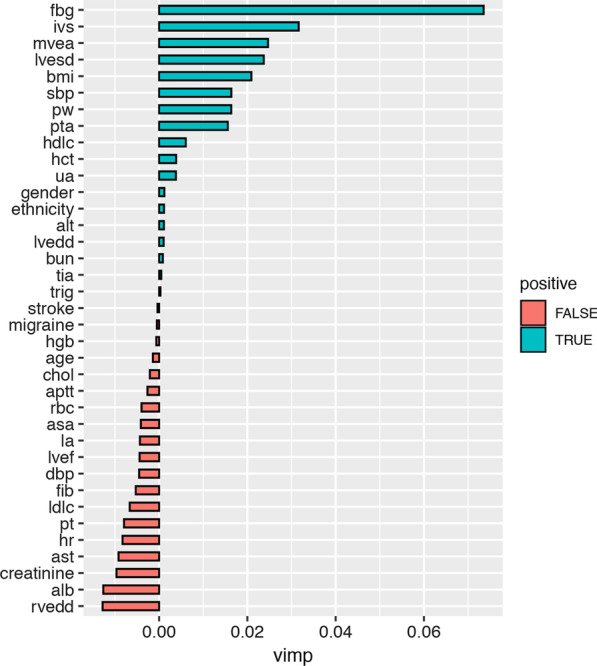
Random forest variable importance (VIMP). Blue bars indicate positive VIMP, red indicates negative VIMP. Importance is relative to positive length of bars

Figure 4 plots the variable importance of the full model using random forest survival analysis. We then selected the eight top ranking features to construct the prediction models, including fasting blood glucose, thickness of interventricular septum, the ratio of mitral peak early (E) to late (A) diastolic filling velocity, left ventricular end-systolic dimension, BMI, systolic blood pressure, thickness of the posterior wall and PTA. As presented in Table 2, the RFS model had similar Brier Score (2.6% *vs* 2.4%) but higher C-index than the traditional Cox proportional hazard regression model (0.87 *vs* 0.54), suggesting a better predictive model for adverse events.

**Table 2:** Performance metrics for different prediction models*

	C-index	95%CI	Brier Score	95%CI
Cox model	0.54	0.20–0.97	0.024	0.007–0.048
RFS model	0.87	0.71–0.98	0.026	0.011–0.046

*Both models were constructed from the top ranking features, including fasting blood glucose, thickness of interventricular septum, the ratio of mitral peak early (E) to late (A) diastolic filling velocity, left ventricular end-systolic dimension, body mass index, systolic blood pressure, thickness of the posterior wall and PTA

*RFS* random forest survival, *CI* confidence incidence

## Discussion

Increasing data have supported that PFO closure could significantly reduce the risk of stroke or TIA compared to medical therapy [7, 9–12]. The reported rate of recurrent stroke or TIA after PFO closure varied across different studies, ranging from 0 to 5.61% [4–10]. As pointed out by previous researchers, the key determination of treatment effect relies mainly on whether the discovered PFO is causally related to the stroke or just an innocent bystander [19]. Currently, there are two prediction systems used to evaluate the likelihood of a stoke-related PFO-the risk of paradoxical embolism (RoPE) score and the PASCAL classification system [14, 20].

Main components for RoPE score include age, smoking status, history of hypertension, diabetes, stroke or TIA, characteristics of the infarct on imaging [20]. PASCAL classification system is based on RoPE score, with combined consideration of PFO features, like PFO shunt size or the presence/absence of an ASA [2, 14]. Although the prediction systems are widely used in clinical practice, these estimations are sometimes violated by model assumptions or limited by subjective feature selections.

Machine learning is a promising technique increasingly applied in health care system. It allows phenotyping or sub-clustering of the analyzed population without knowing the definite outcomes, which helps reveal the underlying etiologies. In this study, we identified two main clusters in post-closure PFO patients, with the high risk cluster characterized by higher proportion of male gender and poorer cardiovascular profiles. Machine learning also enables objective feature selections and efficient prediction model constructions. The analysis of RFS further supported the predictive value of traditional risks factors, suggesting that high-risk groups should continue to be targeted to prevent stroke recurrence even after PFO closure [21, 22]. However, whether maximizing the management of these factors would provide extra benefits for these patients warrants further investigations.

Till now, few studies are conducted on machine learning and PFO [16, 17]. Owing to the small number of adverse outcomes, statistical power to identify independent predictors of recurrent stroke/TIA was often limited, when using the traditional Cox regression model [23]. The application of supervised machine learning to some extent helps settle this matter [24]. As shown in this study, RFS model did display better performance compared to Cox regression model after selecting the top ranking features. Additionally, RFS model was able to identify predictive features that were neglected in previous studies, for example, the ratio of mitral peak early (E) to late (A) diastolic filling velocity and thickness of the interventricular or posterior wall.

Although this is a pioneer study, several limitations should still be acknowledged. First, this is a post-hoc analysis, some data are not available, for example, PFO shunt size before closure or residual shunting after closure. Second, the small datasets and missing data could potentially bias the results, although the missing pattern suggests a random missing data case and the results from complete case analysis was similar to that of the imputed datasets. Third, the constructed model was not further validated by external datasets, which to some extent limits its generalizability. Finally, the evaluation of AF was based on cardiac assessment during follow-up visits. Occult AF might still be possible, leading to an underestimation of the prevalence of AF being reported.

## Conclusions

There were 2 main clusters in PFO patients receiving device closure. The supervised and unsupervised machine learning both suggest that traditional cardiovascular profiles remain important predictors for future recurrence of stroke or TIA. However, whether maximizing the management of these factors would provide extra benefits in post-closure PFO patients warrants further investigations.

## Supplementary Information

**Additional file 1.** **eTable1** Variable summary for missing data. **eTable2** Hierarchical clustering on principal components analysis of the patients. **eTable3** Baseline characteristic of the studied patients across the clusters (k-means clustering analysis). **eTable4** Baseline characteristics of the studied patients across the clusters (in complete case analysis). **eFigure1** Missing data pattern. **eFigure2** Clusters identified by different methods in complete case analysis.

## Data Availability

The datasets and analyzed codes are available upon reasonable request from the corresponding author.

## Abbreviations

PFO: Patent foreman ovale
TIA: Transient ischemic attack
ASA: Atrial septal aneurysm
HCPC: Hierarchical clustering on principal components
RFS: Random forest survival
SBP: Systolic blood pressure
BMI: Body mass index
HDL-C: High-density lipoprotein cholesterol
LDL-C: Low-density lipoprotein cholesterol
