# Supplementary material for: Machine learning in a real-world PFO study: analysis of data from multi-centers in China
Source: BMC Med Inform Decis Mak. 2022 Nov 24;22:305. doi: 10.1186/s12911-022-02048-5 (PMC9694545; doi:10.1186/s12911-022-02048-5)
Supplement: Supplementary file 1 — Additional file 1. eTable1 Variable summary for missing data. eTable2 Hierarchical clustering on principal components analysis of the patients. eTable3 Baseline characteristic of the studied patients across the clusters (k-means clustering analysis). eTable4 Baseline characteristics of the studied patients across the clusters (in complete case analysis). eFigure1 Missing data pattern. eFigure2 Clusters identified by different methods in complete case analysis. [file 12911_2022_2048_MOESM1_ESM.docx]

eTable1 Variable summary for missing data.

|  | Missing | | Valid N | Mean | SD |
| --- | --- | --- | --- | --- | --- |
|  | N | Percent |  |  |  |
| Ethnicity | 1 | 0.51 | 196 | 1.09 | 0.28 |
| Heart rate | 1 | 0.51 | 196 | 77.18 | 11.56 |
| Body mass index | 25 | 12.69 | 172 | 23.35 | 3.25 |
| Red blood cell | 1 | 0.51 | 196 | 4.59 | 0.68 |
| APTT | 10 | 5.08 | 187 | 36.94 | 27.29 |
| PT | 4 | 2.03 | 193 | 13.22 | 2.82 |
| PTA% | 28 | 14.21 | 169 | 71.47 | 42.36 |
| Fibrin | 10 | 5.08 | 187 | 3.12 | 2.05 |
| AST | 5 | 2.54 | 192 | 23.17 | 10.34 |
| ALT | 6 | 3.05 | 191 | 29.52 | 31.43 |
| Albumin | 4 | 2.03 | 193 | 40.73 | 4.73 |
| Creatinine | 2 | 1.02 | 195 | 70.20 | 17.36 |
| BUN | 2 | 1.02 | 195 | 4.98 | 2.76 |
| Uric acid | 18 | 9.14 | 179 | 365.09 | 99.51 |
| Fasting blood glucose | 57 | 28.93 | 140 | 5.06 | 1.27 |
| Triglycerides | 22 | 11.17 | 175 | 1.39 | 0.88 |
| Total cholesterol | 22 | 11.17 | 175 | 4.10 | 1.13 |
| LDL-C | 22 | 11.17 | 175 | 2.39 | 0.83 |
| HDL-C | 22 | 11.17 | 175 | 1.22 | 0.57 |
| Presence of ASA | 20 | 10.15 | 177 | 0.10 | 0.30 |
| LA | 6 | 3.05 | 191 | 32.00 | 4.57 |
| LVEDD | 6 | 3.05 | 191 | 45.31 | 4.67 |
| LVESD | 18 | 9.14 | 179 | 29.33 | 4.51 |
| IVS | 6 | 3.05 | 191 | 9.44 | 1.51 |
| PW | 6 | 3.05 | 191 | 9.22 | 1.25 |
| LVEF | 7 | 3.55 | 190 | 65.85 | 5.41 |
| MVE/MVA | 12 | 6.09 | 185 | 1.12 | 0.34 |

AST, Aspartate aminotransferase; ALT, Alanine aminotransferase; LDL-C, low-density lipoprotein cholesterol; HDL-C, high-density lipoprotein cholesterol; LA, left atrium; LVEDD, left ventricular end-diastolic dimension; LVESD, left ventricular end-systolic dimension; IVS, interventricular septum; PW, posterior wall; LVEF, left ventricular ejection fraction; MVE/MVA, ratio the ratio of mitral peak early (E) to late (A) diastolic filling velocity; ASA, atrial septal aneurysm.

eTable2 Hierarchical clustering on principal components analysis of the patients.

|  | eigenvalue | percentage of variance | cumulative percentage of variance |
| --- | --- | --- | --- |
| comp 1 | 4.70 | 13.82 | 13.82 |
| comp 2 | 3.08 | 9.05 | 22.86 |
| comp 3 | 2.80 | 8.23 | 31.09 |
| comp 4 | 2.22 | 6.54 | 37.63 |
| comp 5 | 2.18 | 6.42 | 44.05 |
| comp 6 | 1.81 | 5.33 | 49.38 |
| comp 7 | 1.50 | 4.41 | 53.80 |
| comp 8 | 1.33 | 3.90 | 57.70 |
| comp 9 | 1.25 | 3.67 | 61.37 |
| comp 10 | 1.08 | 3.19 | 64.56 |
| comp 11 | 1.05 | 3.10 | 67.66 |
| comp 12 | 1.00 | 2.95 | 70.62 |
| comp 13 | 0.96 | 2.84 | 73.46 |
| comp 14 | 0.94 | 2.78 | 76.23 |
| comp 15 | 0.86 | 2.53 | 78.76 |
| comp 16 | 0.82 | 2.41 | 81.17 |
| comp 17 | 0.73 | 2.13 | 83.30 |
| comp 18 | 0.70 | 2.06 | 85.37 |
| comp 19 | 0.64 | 1.88 | 87.25 |
| comp 20 | 0.58 | 1.71 | 88.96 |
| comp 21 | 0.54 | 1.60 | 90.55 |
| comp 22 | 0.49 | 1.44 | 92.00 |
| comp 23 | 0.47 | 1.38 | 93.38 |
| comp 24 | 0.38 | 1.10 | 94.48 |
| comp 25 | 0.33 | 0.96 | 95.45 |
| comp 26 | 0.30 | 0.89 | 96.34 |
| comp 27 | 0.29 | 0.86 | 97.20 |
| comp 28 | 0.24 | 0.72 | 97.92 |
| comp 29 | 0.19 | 0.56 | 98.48 |
| comp 30 | 0.17 | 0.49 | 98.97 |
| comp 31 | 0.13 | 0.38 | 99.35 |
| comp 32 | 0.12 | 0.34 | 99.69 |
| comp 33 | 0.07 | 0.19 | 99.88 |
| comp 34 | 0.04 | 0.12 | 100.00 |

comp: component.

eTable3 Baseline characteristic of the studied patients across the clusters (k-means clustering analysis).

|  | Cluster 1 (n=126) | Cluster 2 (n=71) | Total (n=197) | P value |
| --- | --- | --- | --- | --- |
| Age, years | 43.39(12.08) | 41.80(13.10) | 42.82(12.45) | 0.39 |
| Gender, male | 62(49.21%) | 66(92.96%) | 128(64.97%) | <0.001 |
| Han destiny | 113(89.68%) | 67(94.37%) | 180(91.37%) | 0.26 |
| Heart rate, bpm | 77.57(11.74) | 76.48(11.19) | 77.18(11.53) | 0.53 |
| Systolic BP, mmHg | 120.40(15.86) | 123.92(15.46) | 121.67(15.77) | 0.13 |
| Diastolic BP, mmHg | 76.07(10.28) | 77.70(11.66) | 76.66(10.80) | 0.31 |
| Body mass index, kg/m2 | 22.94(2.76) | 24.06(3.37) | 23.34(3.04) | 0.01 |
| Stroke | 74(58.73%) | 46(64.79%) | 120(60.91%) | 0.40 |
| TIA | 59(46.83%) | 26(36.62%) | 85(43.15%) | 0.17 |
| Migraine | 17(13.49%) | 10(14.08%) | 27(13.71%) | 0.91 |
| laboratory data |  |  |  |  |
| Red blood cell, 10*12/L | 4.46(0.71) | 4.80(0.55) | 4.59(0.68) | <0.001 |
| Hemoglobin, g/dl | 128.92(19.84) | 140.40(14.91) | 133.05(19.00) | <0.001 |
| Hemotocrit | 39.25(5.47) | 41.75(3.83) | 40.15(5.08) | <0.001 |
| APTT | 34.80(5.78) | 40.73(43.55) | 36.94(26.58) | 0.13 |
| PT | 13.03(2.62) | 13.55(3.06) | 13.22(2.79) | 0.21 |
| PTA% | 69.81(39.95) | 74.42(37.98) | 71.47(39.22) | 0.43 |
| Fibrin | 3.14(2.42) | 3.08(0.87) | 3.12(2.00) | 0.84 |
| AST | 22.36(10.75) | 24.61(9.06) | 23.17(10.21) | 0.14 |
| ALT | 25.10(21.11) | 37.38(42.29) | 29.52(30.95) | 0.007 |
| Albumin, g/dl | 40.81(5.24) | 40.6(3.49) | 40.73(4.68) | 0.77 |
| Creatinine | 64.95(14.85) | 79.51(17.42) | 70.20(17.27) | <0.001 |
| BUN | 4.95(3.30) | 5.04(1.31) | 4.98(2.75) | 0.83 |
| Uric acid, | 308.18(54.32) | 466.07(61.16) | 365.09(94.84) | <0.001 |
| Fasting blood glucose, mg/dL | 5.03(1.17) | 5.11(0.86) | 5.06(1.07) | 0.63 |
| Triglycerides, mg/dL | 1.31(0.85) | 1.53(0.77) | 1.39(0.83) | 0.07 |
| Total cholesterol, mg/dL | 4.15(1.02) | 4.00(1.14) | 4.10(1.07) | 0.35 |
| LDL-C, mg/dL | 2.39(0.77) | 2.38(0.82) | 2.39(0.79) | 0.94 |
| HDL-C, mg/dL | 1.22(0.35) | 1.21(0.76) | 1.22(0.54) | 0.84 |
| Echocardiography |  |  |  |  |
| Presence of atrial septal aneurysm | 11(8.73%) | 6(8.45%) | 17(8.63%) | 0.95 |
| LA, mm | 31.80(4.21) | 32.36(4.98) | 32.00(4.50) | 0.41 |
| LVEDD, mm | 45.20(3.28) | 45.50(6.33) | 45.31(4.60) | 0.66 |
| LVESD, mm | 29.34(3.99) | 29.31(4.81) | 29.33(4.29) | 0.96 |
| IVS, mm | 9.29(1.49) | 9.69(1.46) | 9.44(1.49) | 0.07 |
| PW, mm | 9.06(1.23) | 9.51(1.19) | 9.22(1.23) | 0.01 |
| LVEF, % | 65.66(5.14) | 66.19(5.61) | 65.85(5.31) | 0.50 |
| MVE/MVA | 1.12(0.34) | 1.14(0.31) | 1.12(0.33) | 0.71 |
| RVEDD, mm | 29.60(9.17) | 35.15(46.25) | 31.60(28.72) | 0.19 |

TIA, transient ischemic attack; AST, Aspartate aminotransferase; ALT, Alanine aminotransferase; LDL-C, low-density lipoprotein cholesterol; HDL-C, high-density lipoprotein cholesterol; LA, left atrium; LVEDD, left ventricular end-diastolic dimension; LVESD, left ventricular end-systolic dimension; IVS, interventricular septum; PW, posterior wall; LVEF, left ventricular ejection fraction; RVEDD, right ventricular end-diastolic dimension.

eTable4 Baseline characteristics of the studied patients across the clusters (in complete case analysis).

|  | Cluster 1 (n=30) | Cluster 2 (n=27) | Total (n=57) | P value |
| --- | --- | --- | --- | --- |
| Age, years | 38.37(12.61) | 44.44(13.37) | 41.25(13.22) | 0.08 |
| Gender, male | 27(90.0%) | 7(25.93%) | 34(59.65%) | <0.001 |
| Han destiny | 28(93.33%) | 21(77.78%) | 49(85.96%) | 0.09 |
| Heart rate, bpm | 79.73(11.80) | 75.74(12.04) | 77.84(11.98) | 0.21 |
| Systolic BP, mmHg | 120.97(11.64) | 117.48(17.19) | 119.32(14.51) | 0.37 |
| Diastolic BP, mmHg | 78.27(8.33) | 72.96(9.39) | 75.75(9.16) | 0.03 |
| Body mass index, kg/m2 | 24.56(3.88) | 21.46(2.58) | 23.09(3.65) | <0.001 |
| Stroke | 17(56.67%) | 13(48.15%) | 30(52.63%) | 0.52 |
| TIA | 13(43.33%) | 14(51.85%) | 27(47.37%) | 0.52 |
| Migraine | 2(6.67%) | 13(48.15%) | 15(26.32%) | <0.001 |
| laboratory data |  |  |  |  |
| Red blood cell, 10*12/L | 5.01(0.50) | 4.00(0.41) | 4.53(0.68) | <0.001 |
| Hemoglobin, g/dl | 140.40(14.03) | 115.77(15.50) | 128.74(19.17) | <0.001 |
| Hemotocrit | 42.34(3.48) | 36.35(5.04) | 39.51(5.21) | <0.001 |
| APTT | 36.66(4.72) | 34.22(4.90) | 35.51(4.92) | 0.06 |
| PT | 13.48(2.61) | 14.12(3.45) | 13.78(3.03) | 0.43 |
| PTA% | 97.43(18.60) | 55.16(41.50) | 77.41(37.84) | <0.001 |
| Fibrin | 3.05(0.87) | 3.99(3.82) | 3.49(2.72) | 0.19 |
| AST | 25.18(8.31) | 21(7.83) | 23.2(8.28) | 0.06 |
| ALT | 34.27(19.15) | 20.77(9.87) | 27.88(16.78) | 0.002 |
| Albumin, g/dl | 40.94(2.71) | 38.15(4.14) | 39.62(3.70) | 0.004 |
| Creatinine | 80.90(16.07) | 60.38(9.98) | 71.18(16.93) | <0.001 |
| BUN | 4.83(1.24) | 6.38(6.27) | 5.56(4.43) | 0.19 |
| Uric acid, | 419.72(91.96) | 313.02(80.61) | 369.18(101.42) | <0.001 |
| Fasting blood glucose, mg/dL | 5.39(1.86) | 4.81(0.65) | 5.11(1.44) | 0.13 |
| Triglycerides, mg/dL | 1.31(0.77) | 1.46(1.41) | 1.38(1.11) | 0.63 |
| Total cholesterol, mg/dL | 3.93(1.08) | 4.24(1.19) | 4.08(1.13) | 0.31 |
| LDL-C, mg/dL | 2.47(0.93) | 2.15(0.58) | 2.32(0.79) | 0.14 |
| HDL-C, mg/dL | 1.02(0.20) | 1.69(1.15) | 1.34(0.87) | 0.003 |
| Echocardiography |  |  |  |  |
| Presence of atrial septal aneurysm | 3(10%) | 0 | 3(5.26%) | 0.09 |
| LA, mm | 31.2(4.09) | 30.96(3.94) | 31.09(3.98) | 0.82 |
| LVEDD, mm | 45.67(3.49) | 43.33(4.42) | 44.56(4.09) | 0.03 |
| LVESD, mm | 27.23(3.28) | 31.96(5.65) | 29.47(5.11) | <0.001 |
| IVS, mm | 9.68(1.33) | 8.48(0.98) | 9.11(1.31) | <0.001 |
| PW, mm | 9.46(1.13) | 8.39(0.79) | 8.95(1.12) | <0.001 |
| LVEF, % | 68.2(5.56) | 66.89(5.41) | 67.58(5.48) | 0.37 |
| MVE/MVA | 1.15(0.36) | 1.23(0.29) | 1.19(0.32) | 0.35 |
| RVEDD, mm | 29.87(14.01) | 32.04(10.85) | 30.89(12.55) | 0.52 |

TIA, transient ischemic attack; AST, Aspartate aminotransferase; ALT, Alanine aminotransferase; LDL-C, low-density lipoprotein cholesterol; HDL-C, high-density lipoprotein cholesterol; LA, left atrium; LVEDD, left ventricular end-diastolic dimension; LVESD, left ventricular end-systolic dimension; IVS, interventricular septum; PW, posterior wall; LVEF, left ventricular ejection fraction; RVEDD, right ventricular end-diastolic dimension.

eFigure1 Missing data pattern. There is no particular trend among all the variables with regard to their missing data. It is considered as a random missing data case.

eFigure2 Clusters identified by different methods in complete case analysis. (A) Dendrogram from hierarchical clustering on principal components analysis. (B) The average silhouette of observations for different values of k (1 to 10) using k-means clustering analysis. The highest average silhouette was located at k=2.

**R Source Code:**

**########################Unsupervised Machine Learning#####################**

**# Hierarchical Clustering on Principal Components Analysis**

#Compute PCA

library(FactoMineR)

set.seed(1234)

res<-FAMD(pfo,graph=FALSE)

res.hcpc<-HCPC(res,graph=FALSE)

res.hcpc$data.clust$clust

#Visualization

fviz_dend(res.hcpc,

cex = 0.7,

palette = "jco",

rect = TRUE, rect_fill = TRUE,

rect_border = "jco",

labels_track_height = 0.8)

# Visualize individuals on the principal component map and to color individuals according to the cluster they belong to.

fviz_cluster(res.hcpc,

repel = TRUE,

show.clust.cent = TRUE,

palette = "jco",

ggtheme = theme_minimal(),

main = "Factor map")

# Inspect eigenvalues

res$eig

# Visualize variance

fviz_eig(res, addlabels = TRUE)

**# K-MEANS CLUSTERING**

# CHOOSING K

library(factoextra)

library(NbClust)

fviz_nbclust(pfo, kmeans, method = "silhouette")+

labs(subtitle = "Silhouette method")

### K-means clustering with optimal clusters K=2

fitKFAcluster <- kmeans(pfo, 2)

fitKFAcluster

fitKFAcluster$cluster

**############################Supervised Machine Learning######################**

**# Supervised Self-Organizing Maps**

library(kohonen)

library(dplyr)

# Data Split

set.seed(123)

ind <- sample(2, nrow(data), replace = T, prob = c(0.7, 0.3))

train <- data[ind == 1,]

test <- data[ind == 2,]

# Normalization

trainX <- scale(train)

testX <- scale(test,

center = attr(trainX, "scaled:center"),

scale = attr(trainX, "scaled:scale"))

trainY <- factor(train[,1])

Y <- factor(test[,1])

test[,1] <- 0

testXY <- list(independent = testX, dependent = test[,1])

# Classification & Prediction Model

set.seed(222)

map1 <- xyf(trainX,

classvec2classmat(factor(trainY)),

grid = somgrid(5, 5, "hexagonal"),

rlen = 100)

plot(map1)

# Prediction

pred <- predict(map1, newdata = testXY)

table(Predicted = pred$predictions[[2]], Actual = Y)

# Cluster Boundaries

par(mfrow = c(1,2))

plot(map1,

type = 'codes',

main = c("Codes X", "Codes Y"))

map1.hc <- cutree(hclust(dist(map1$codes[[2]])), 2)

add.cluster.boundaries(map1, map1.hc)

par(mfrow = c(1,1))

**#Random survival forest and variable importance analysis**

library(survival)

library(randomForestSRC)

library(riskRegression)

library(ggRandomForests)

set.seed(123)

rfsrc<-rfsrc(Surv(time, event)~., data=pfo, na.action="na.impute",nsplit = 10,

tree.err = TRUE,

importance = TRUE)

rfsrc

plot(gg_vimp(rfsrc))

###Comparisons between models

#Cox proportional hazards regression model

fit.cox <-coxph(Surv(time,event)~fbg+ivs+mvea+lvesd+bmi+sbp+pw+pta, pfo,x=T)

#Random survival Forest model

fit.rsf <-rfsrc(Surv(time,event)~fbg+ivs+mvea+lvesd+bmi+sbp+pw+pta, data=pfo,nsplit = 10,

tree.err = TRUE, na.action=”na.impute”,

importance = TRUE)

#Model comparisons~C-index and Brier Score

fitcomp<- riskRegression::Score(list("COX"=fit.cox,

"RSF"=fit.rsf),

formula=Hist(time,event)~fbg+ivs+mvea+lvesd+bmi+sbp+pw+pta,

data=pfo, metrics = c("auc", "brier"),

split.method="bootcv",B=5)

**Source Code in Stata 15.1 (StataCorp/SE, College Station, TX)**

**# Cox Proportional Hazard Regression Analysis**

stset days_fu, failure(adverseoutcome)

stcox clust_hcpc

estat phtest

sts test clust_hcpc

sts graph, cumhaz by(clust_hcpc)

stcox clust_kmeans

estat phtest

sts test clust_kmeans

sts graph, cumhaz by(clust_kmeans)
